# Functional and Physiological Characterization of Tyrosine Decarboxylases from *Olea europaea* L. Involved in the Synthesis of the Main Phenolics in Olive Fruit and Virgin Olive Oil

**DOI:** 10.3390/ijms252010892

**Published:** 2024-10-10

**Authors:** Pilar Luaces, Rosario Sánchez, Jesús Expósito, Antonio J. Pérez-Pulido, Ana G. Pérez, Carlos Sanz

**Affiliations:** 1 Department of Biochemistry and Molecular Biology of Plant Products, Instituto de la Grasa, Spanish National Research Council (CSIC), 41013 Seville, Spain; pluaces@ig.csic.es (P.L.); rsanchez@ig.csic.es (R.S.); jexposito@ig.csic.es (J.E.); 2 Andalusian Centre for Developmental Biology (CABD, UPO-CSIC-JA), Faculty of Experimental Sciences (Genetics Area), University Pablo de Olavide, 41013 Seville, Spain; ajperez@upo.es

**Keywords:** *Olea europaea*, hydroxytyrosol, tyrosol, tyrosine decarboxylase, olive fruit, phenolics, quality, virgin olive oil

## Abstract

The phenolic composition of virgin olive oil (VOO) primarily depends on the phenolic content of the olive fruit. The purpose of this work was to characterize the first metabolic step in the synthesis of tyrosol (Ty) and hydroxytyrosol (HTy), whose derivatives are by far the predominant phenolics in both olive fruit and VOO. To this end, two genes encoding tyrosine/DOPA decarboxylase enzymes, *OeTDC1* and *OeTDC2*, have been identified and functionally and physiologically characterized. Both olive TDC proteins exclusively accept aromatic amino acids with phenolic side chains, such as tyrosine and 3,4-dihydroxyphenylalanine (DOPA), as substrates to produce tyramine and dopamine, respectively. These proteins exhibited a higher affinity for DOPA than for tyrosine, and the catalytic efficiency of both proteins was greater when DOPA was used as a substrate. Both olive *TDC* genes showed a fairly similar expression profile during olive fruit ontogeny, with *OeTDC1* consistently expressed at higher levels than *OeTDC2*. Expression was particularly intense during the first few weeks after fruit set, coinciding with the active accumulation of Ty and HTy derivatives. The data suggest that both olive TDCs are responsible for the initial step in the synthesis of the most important phenolics, both quantitatively and functionally, in VOO.

## 1. Introduction

Virgin olive oil (VOO), one of the oldest known plant oils, is the primary source of lipids in the Mediterranean diet and is a major contributor to the well-documented health benefits associated with this diet. Prolonged consumption of VOO may attenuate the inflammatory response in the body, thereby reducing the risk of suffering from any of the numerous diseases related to chronic inflammation [1]. Special attention has been given to the phenolic fraction, which is primarily responsible for the health benefits of VOO and is currently used as a quality marker for VOO [2,3,4]. The phenolic composition of VOO largely depends on the phenolic content of the olive fruit [5], which has recently been used as a trait in olive breeding programs [6]. Olive fruit phenolics mainly comprise glycosylated derivatives of secoiridoid compounds that include tyrosol (Ty) and hydroxytyrosol (HTy) residues in their molecules. These simple alcohols appear to be the only compounds present in the bloodstream after virgin olive oil (VOO) consumption and are, therefore, likely the primary agents responsible for the health benefits of olive oil [4]. Additionally, flavonoids, which are mainly composed of flavones and anthocyanins, as well as tocopherols, are present but in much smaller proportions than secoiridoids [5,7]. The main phenolic glycosides of olive fruit are oleuropein, ligstroside and demethyloleuropein [5,8]. Their enzymatic hydrolysis during the oil extraction process results in the formation of the majority of phenolics found in VOO [9].

**Figure 1:**
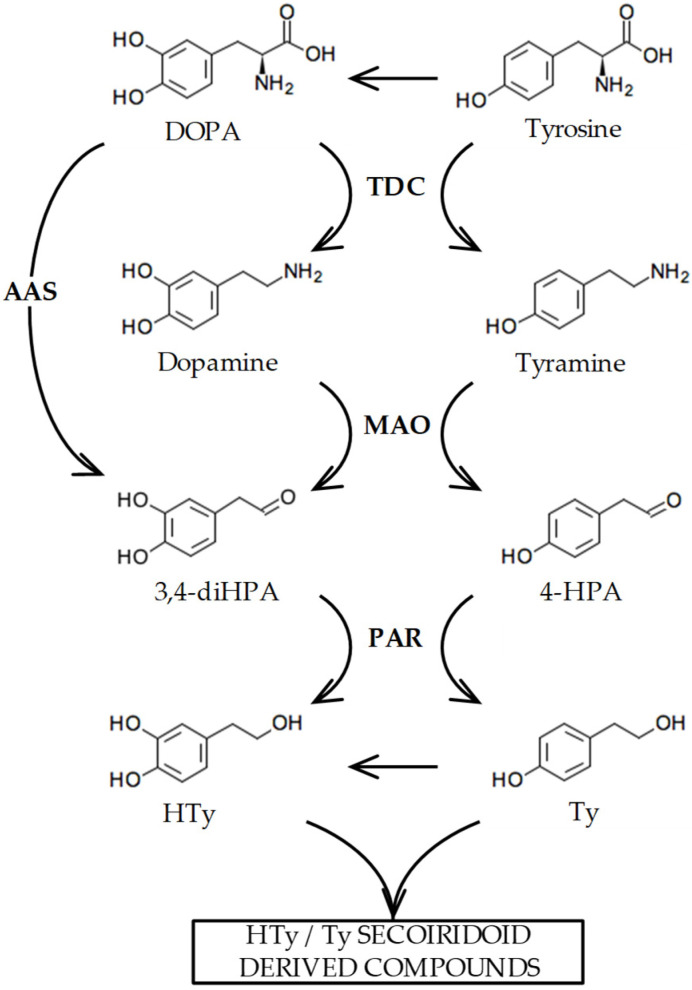
Biosynthesis pathway proposed for the phenolic alcohols hydroxytyrosol (HTy) and tyrosol (Ty) in olive [10]. The abbreviated intermediate metabolites and enzymes are as follows: 3,4 dihidroxyphenylacetaldehyde (3,4-diHPA); 4-hydroxyphenylacetaldehyde (4-HPA); tyrosine decarboxylase (TDC); monoamino oxidase (MAO); aromatic aldehyde synthase (AAS); phenylacetaldehyde reductase (PAR).

Figure 1 summarizes the biosynthesis of Ty and HTy that we have previously suggested may occur in olive [10] based on the evidence found in other plant species [11]. These compounds are generated from tyrosine and 3,4-dihydroxyphenylalanine (DOPA) through sequential processes including amino acid decarboxylation, primary amine oxidative deamination, and aldehyde reduction. Although the hypothesized biosynthetic pathway of Ty and HTy seem reasonable, to date, enzymes or their coding genes involved in the decarboxylation and deamination in this pathway have not been fully characterized. In this regard, coupling metabolic and transcriptomic data with protein functional characterization enabled us to identify the *OeAAS* gene, whose encoded protein (*Oe*AAS, EC 4.1.1.107) is a highly specific enzyme that catalyzes the conversion of DOPA into 3,4-dihydroxyphenylacetaldehyde (3,4-diHPA) in a single step, the aldehyde precursor of HTy [10]. The catalytic properties of *Oe*AAS and the expression pattern of its coding gene in different olive cultivars with contrasting phenolic contents suggest that *Oe*AAS may have a key role in the accumulation of HTy-derived secoiridoids during olive fruit ripening. However, the dynamics of phenolic content during the ontogeny of the olive fruit point to very active synthesis of these compounds in the first few weeks after fruit set followed by a subsequent decrease until the end of the fruit ripening phase is reached [12]. These data suggest the possibility that the decarboxylation and oxidative deamination steps to produce the aldehyde precursors of Ty and HTy were fully active in those first few weeks of fruit development.

The decarboxylation of aromatic amino acids is apparently ubiquitous in the plant kingdom, playing an important role in different metabolic pathways [13] by providing key precursors for the biosynthesis of different classes of plant natural products such as indole alkaloids, benzylisoquinoline alkaloids, hydroxycinnamic acid amides, phenylacetaldehyde-derived floral volatiles, and tyrosol derivatives. Tyrosine decarboxylases (TyDC, EC 4.1.1.25) are proteins that belong to the pyridoxal 5′-phosphate (PLP)-dependent aromatic amino acid decarboxylase (AAAD) family, which has yielded an array of paralogous enzymes exhibiting divergent substrate preferences and catalytic mechanisms. TyDC catalyzes the decarboxylation of tyrosine and DOPA [14,15,16]. Plant *TyDC* genes were found to encompass small gene families in several species [17,18]. In olive, the existence of a functional TyDC was demonstrated by Saimaru and Orihara [19], who observed the conversion of labeled DOPA into dopamine in olive cell cultures. However, to our knowledge, no information is currently available regarding the genes encoding these proteins in olive.

The aim of this work is to characterize the genes responsible for the biosynthesis of Ty and HTy derivatives in the olive fruit, focusing on the first metabolic step in this process—the decarboxylation of aromatic amino acids—at molecular, physiological, and catalytic levels, to determine their role in the biosynthesis of Ty and HTy in olive fruit. These derivatives constitute the primary phenolic compounds in VOO, both quantitatively and, more importantly, functionally, as they are chiefly responsible for its health-promoting properties. Therefore, characterizing the genes involved in their synthesis could be highly beneficial for identifying key molecular markers and genetic variants that influence phenol content and composition in marker-assisted olive breeding programs.

## 2. Results and Discussion

### 2.1. Identification and Molecular Characterization of Olive TDC Genes

Five genes annotated as tyrosine/DOPA decarboxylases were identified in the transcriptome of seven olive cultivars characterized by their contrasting phenolic compound contents (Table S1) [10]. Based on the proposed biosynthetic pathway shown in Figure 1, it is presumed that their encoded proteins could be involved in the first step of the biosynthesis of Ty and HTy derivatives in olive. Only three of them (Olive Genome Database accession OE6A000744, OE6A073377 and OE6A082511) exhibited relevant expression levels during fruit ripening and significant differential expression among the olive cultivars and ripening stages used in the generation of these genomic tools. Although all of their encoded proteins were annotated as tyrosine/DOPA decarboxylases, we previously found that the *OE6A000744* gene actually encodes an aromatic aldehyde synthase (*Oe*AAS, GenBank Accession number QJA07379.1), since the recombinant protein was shown to be a bifunctional enzyme that catalyzes decarboxylation and dependent oxidative deamination reactions in a single step [10]. Given that the annotations are in some cases incorrect, the other two decarboxylases, named *OeTDC1* (OE6A082511, GenBank accession number PP534480) and *OeTDC2* (OE6A073377, GenBank accession number PP590793), respectively, have been studied at the molecular and biochemical levels to determine if their properties are consistent with their potential role in the synthesis of the Ty and HTy derivatives present in VOO.

**Figure 2:**
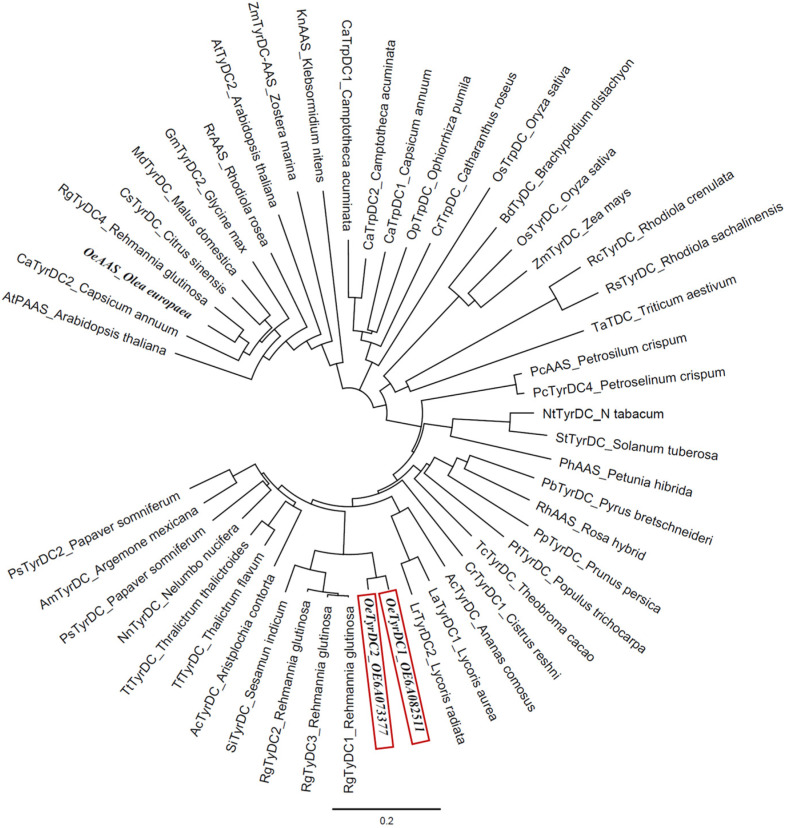
Phylogenetic tree illustrating the relation of olive TDCs (framed in red) to other plant AAADs. Accession numbers of the different AAS/TyDC included in the analysis are shown in Table S2.

A phylogenetic analysis was conducted, including the two olive TDC homologs and the *Oe*AAS sequences together with other AAAD sequences from taxonomically diverse plant species (Figure 2). The two olive TDC proteins fall into a clade largely composed of previously known TyDCs, referred to as subset I by Yang et al. [20], which include *Rg*TyDC1-3 from *Rehmannia glutinosa*, *La*TyDC from *Lycoris aurea* and *Ps*TyDCs from *Papaver somniferum*, and this clade is distinct from subset II, which exhibited the closest relationship with *Rg*TyDC4 from *R. glutinosa*, *Md*TyDC from *Malus domestica*, *At*PAAS from *Arabidopsis thaliana* and several AASs, including *Oe*AAS. The olive TDCs group together, sharing 95% amino acid identity. They share a greater proportion of amino acid identity with the protein from *R. glutinosa* (*Rg*TyDC1 86%) than with other previously characterized TyDC proteins, such as those from *P. somniferum* (*Ps*TYDC2, 76%), *Catharanthus roseus* (*Cr*TDC 56%) or *Oryza sativa* (*Os*TyrDC, 51%).

*OeTDC1* and *OeTDC2* are genes that are 2370 and 2174 bp long and contain coding regions of 1524 bp for both; these regions are larger than the coding region of the *OeAAS* gene (1464 bp). The predicted *Oe*TDC proteins exhibited similar lengths (507 amino acid residues) and slightly different calculated molecular weights: 56.3 and 56.4 KDa, and isoelectric points, 6.65 and 6.48, for *Oe*TDC1 and *Oe*TDC2, respectively. These values differ significantly from those of the *Oe*AAS protein, having 487 amino acid residues, a calculated molecular mass of 53.9 kDa and an isoelectric point of 6.03 [10]. The subcellular localization prediction program DeepLoc2.0 [21] indicated a greater probability of cytoplasmic localization for both TDCs and TargetP-2.0 [22] did not identify signal peptides at their N termini for any of them. Similarly, analysis of the *Oe*AAS protein sequence with these targeting and localization prediction tools did not show signal peptides and a cytosolic localization for the protein, respectively, similar to the findings reported for other AAS proteins [23].

**Figure 3:**
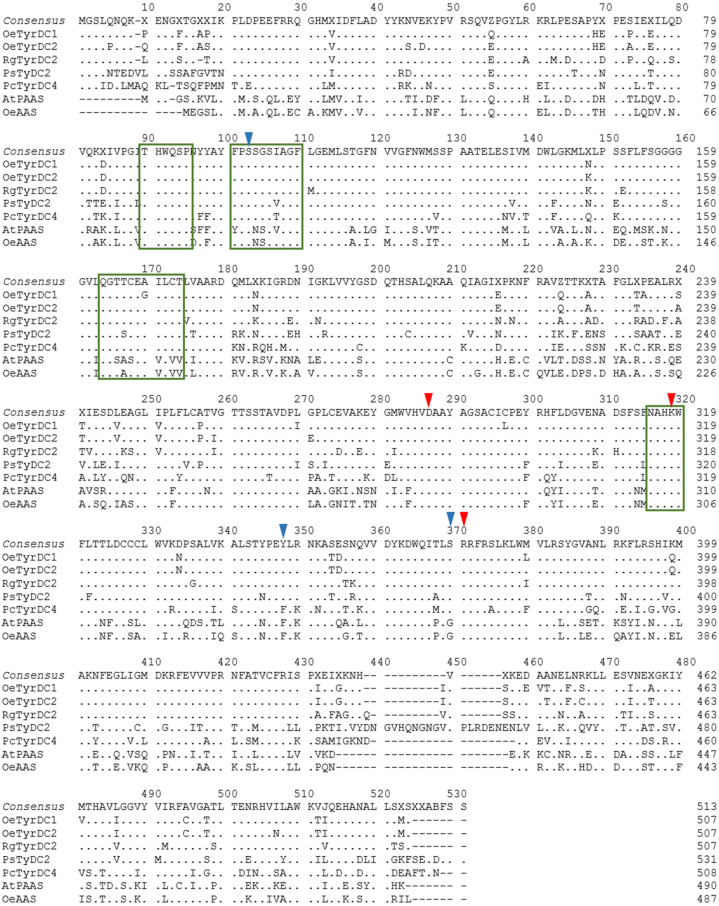
Amino acid sequence alignment of olive TDCs and other AAAD proteins with demonstrated functionality as tyrosine decarboxylases and aromatic aldehyde synthase. The identical residues are plotted by dots. Hyphens indicate gaps in the alignment. Highly conserved domains are marked with green boxes. Red arrows indicate amino acids involved in the pyridoxal 5′-phosphate (PLP) binding site: the PLP-binding lysine residue (K^318^), the aspartate D^286^ that stabilizes the protonated form of the pyridine nitrogen of PLP bound to K^318^, and the arginine residue at position 370 (R^370^) that recognizes the carboxylic group of the substrate. Blue arrows mark amino acids involved in activity and substrate specificities: the residue dictating activity type at position 347; the amino acid involved in the substrate specificity at position 369; and the residue at position 102 that dictates the affinity for hydroxylated/non-hydroxylated substrates. The GenBank accession number for each sequence is listed in Table S2 in the Supplementary Materials.

The deduced amino acid sequences of the two *Oe*TDCs and *Oe*AAS [10] and the previously characterized TyDCs and AASs proteins were aligned together (Figure 3) and subjected to the NCBI Conserved Domain Search to identify sequence motifs that determine the different catalytic mechanisms exhibited by the enzymes of the AAAD family [24,25].

The predicted *Oe*TDC proteins retain a set of conserved domains typical of plant AAAD proteins and group II PLP-dependent amino acid decarboxylases, including key amino acids involved in the PLP binding site, such as the PLP-binding lysine residue at the catalytic active site (K^318^ in the olive TDC sequences) [16]. The alignment showed that the *Oe*TDC proteins also retained substrate specificity sites, which are known to be substrate-binding domains [15,26,27] such as a tyrosine residue at position 347 (Y^347^) in the olive TDC sequences (Figure 3) associated with decarboxylating activity [25]. In contrast, the AAS proteins display a phenylalanine residue at the same position, associated with the bifunctional catalytic activity of these enzymes, which includes both decarboxylation and dependent oxidative deamination of the aromatic amino acids [24]. Furthermore, olive TDCs also show a serine at position 369 (S^369^), which seems to be involved in the indolic or phenolic substrate specificity [25], indicating that these proteins decarboxylate primarily aromatic amino acids with phenol side chains; and also a serine (S^102^) that dictates the affinity for hydroxylated substrates, compared to the alternative alanine in this position (A^102^), which is associated with non-hydroxylated substrates [28]. Other possible roles for residues in AAADs have been suggested by Ishii et al. [29]. Thus, the arginine at position 370 (R^370^), which is absolutely conserved in all AAAD sequences, might be necessary for the recognition of the carboxylic group of the substrate. Furthermore, the aspartate at position 286 (D^286^) is invariant among AAADs. The negatively charged carboxylate group seems to be essential for the stabilization of the protonated form of the pyridine nitrogen of PLP bound to K^318^ [28]. These results suggest that the olive TDC proteins possess potential functional characteristics similar to those coded by homologous TyDC genes from other plants.

### 2.2. Catalytic Properties of the Recombinant Olive TDC Proteins

Based on the in silico analyses, *OeTDC1* and *OeTDC2* were identified as encoding TyDC proteins. To explore the potential role of the olive *TDC* genes in the biosynthesis of Ty and HTy derivatives in olives, the full-length open reading frames corresponding to *OeTDC1* and *OeTDC2* were synthesized, cloned and expressed as recombinant proteins in *E. coli* as detailed in Section 3. The *E. coli* culture conditions were optimized for slow growth (18 h) at low temperature (18 °C) in LB medium in order to enhance the solubility of the expressed proteins. Under these conditions, the recombinant proteins were detected in the soluble fractions of *E. coli* cells as a major band of the expected size after SDS-PAGE analysis (Figure S1). The molecular weights calculated by SDS-PAGE analysis for both *Oe*TDC1 and *Oe*TDC2 proteins were around 57 kDa, closely matching the theoretical values predicted from the amino acid sequences, including the six histidines added to each construction. These weights were consistent with those of similar enzymes in other plant species, such as those from *R. glutinosa* [20], *Eschscholzia californica* or *Thalictrum rugosum* [30].

**Figure 4:**
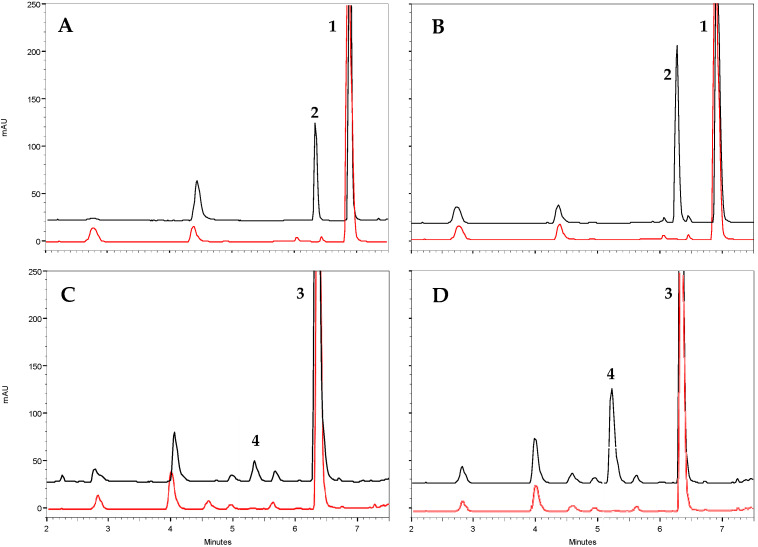
Functional characterization of the purified recombinant olive TDC proteins by in vitro enzyme assays. Representative HPLC analysis (black trace) of the reaction of *Oe*TDC1 and *Oe*TDC2 toward tyrosine (**A** and **B**, respectively) and toward DOPA (**C** and **D**, respectively). The peaks in the chromatograms represent 1, tyrosine (R_t_ = 6.9 min); 2, tyramine (R_t_ = 6.3 min); 3, DOPA (R_t_ = 6.4 min); and 4, dopamine (R_t_ = 5.2 min). Control reactions (red trace) used protein deactivated at 100 °C for 1 h.

Regarding catalysis, both olive TDC proteins exhibited optimal activity at a pH of around 8 and a temperature close to 40 °C (Figure S2). These values are similar to those observed in other plant species [23,25,31]. Under these optimal conditions, the type of reaction catalyzed by these proteins was studied through HPLC-DAD/MS analysis. It was found that both proteins exhibited decarboxylase activity, producing dopamine and tyramine from DOPA and tyrosine, respectively (Figure 4). No evidence of 3,4-diHPA or 4-HPA production was detected in the reactions, indicating that *Oe*TDC1 and *Oe*TDC2 can be considered strict decarboxylases, which are apparently devoid of any residual AAS-type activity. These findings are consistent with the molecular properties, as both have a tyrosine at position 347 (Y^347^), which determines this reaction type within the AAAD family.

Likewise, the amino acid determinants that seem to be involved in substrate recognition and that are present in both proteins (S^102^ and S^369^) align with the results obtained. The specificity for different aromatic amino acids was tested in vitro and it was found that both proteins exhibited activity with the phenolic amino acids DOPA and tyrosine but had no activity with amino acids possessing an indolic (tryptophan) or non-hydroxylated-phenyl (phenylalanine) structure, as reported for all plant tyrosine decarboxylases [29]. *Oe*TDC1 and *Oe*TDC2 demonstrated specific activities of 206 and 425 nkat mg^−1^ towards tyrosine, and 68 and 257 nkat mg^−1^ towards DOPA, respectively (Table 1, Figure S3). The calculated *k***_cat_** values were 12.79 and 23.98 s^−1^ for tyrosine and 3.82 and 14.50 s^−1^ for DOPA, respectively. Regarding substrate affinity, the *K*m values for the *Oe*TDC1 and *Oe*TDC2 proteins were 1.90 and 1.29 mM for tyrosine and 0.34 and 0.46 mM for DOPA, respectively. All reported plant tyrosine decarboxylases accept tyrosine and DOPA as substrates, with the relative activity towards these substrates being dependent on the plant species [29]. Thus, parsley TDC shows a high preference for tyrosine (3-to-1 over DOPA), while, on the contrary, sanguinaria TDC has a greater affinity for DOPA (30-to-1 over tyrosine). The calculation of the catalytic efficiency indicated that *Oe*TDC2 exhibits higher efficiency than *Oe*TDC1, and for both proteins, the efficiency is greater when DOPA is used as substrate (Table 1). The *K*m values were very similar to most of those found for other plant TDCs or AASs such as poppy [25,26], rhodiola [31], arabidopsis [32], parsley [33] or petunia [23]. On the other hand, olive TDCs appear to have a slightly lower affinity for DOPA than that found for *Oe*AAS (*K*m = 0.15 mM) [10]. The difference in *K*m values becomes even greater when the calculations are performed under the optimal reaction conditions for *Oe*AAS (pH 6.8 and 30 °C). Under these conditions, the calculated *K*m values showed that the affinity towards DOPA is lower for both proteins (*Oe*TDC1, *K*m = 2.99 mM; *Oe*TDC2, *K*m = 2.63 mM).

Finally, it was observed that PLP binds tightly to the olive TDC proteins. PLP is incorporated into the protein during its synthesis and folding process. No enzymatic activity was detected in the absence of PLP during the extraction and purification of the olive TDC proteins, and the addition of PLP to purified, PLP-free preparations did not restore activity. Additionally, the presence of PLP in the reaction medium was not required during the measurement of enzymatic activity in fully functional olive TDC proteins. In contrast, results for a TDC from *Papaver somniferum* indicated that this protein weakly binds the PLP cofactor [16]. A similar observation was made with *Oe*AAS, which also exhibited weaker PLP binding, a characteristic that appears to be common among all plant AASs [23,24,31].

### 2.3. Expression of TDC Genes along the Olive Fruit Ontogeny

**Figure 5:**
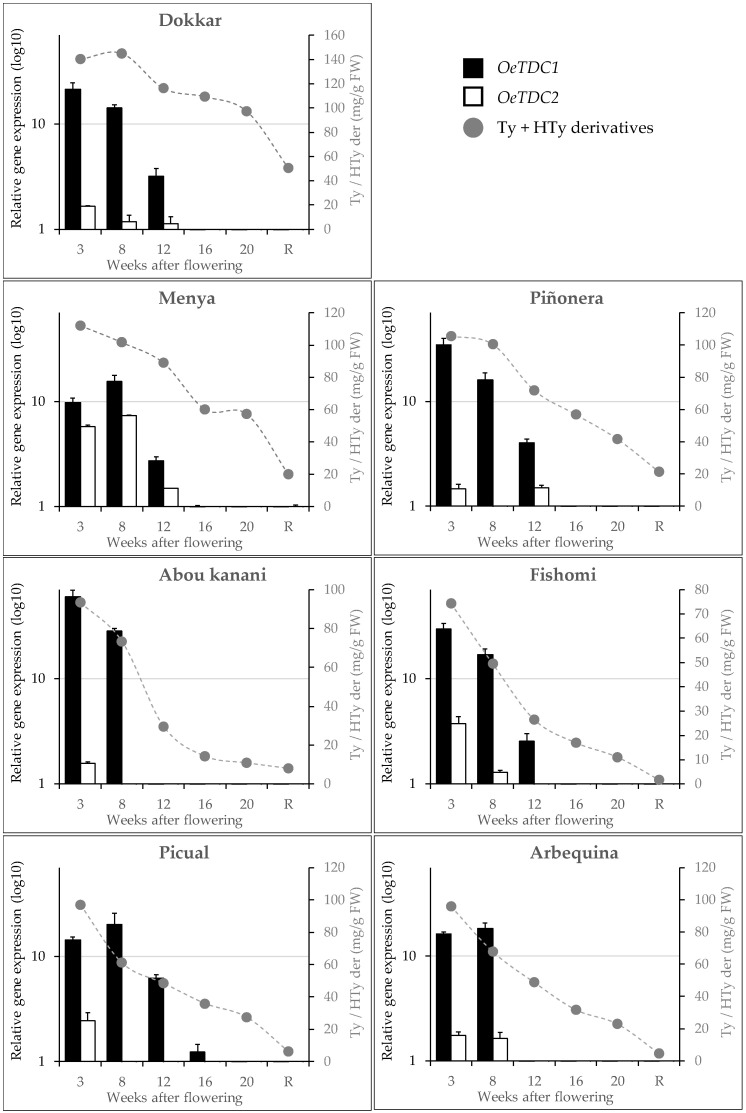
Relative expression levels (log10) of olive *TDC* genes in the mesocarp tissue of olive fruits along fruit ontogeny (fruits at 3 to 20 weeks after flowering and ripe fruits, R). Data are mean ± SD. Three biological and two technical replicates were obtained for each sample. Content of phenolics derivatives of tyrosol (Ty) and hydroxytyrosol (Hty) (mg g^−1^ FW) according to Luaces et al. [12].

We have recently found in different olive cultivars [12] that, unlike the pattern followed by flavonoids or tocopherols, just after fruit set, there is an intense accumulation of tyrosol and hydroxytyrosol derivatives in the fruit, as well as a subsequent constant decrease in the content of these phenols throughout the ontogeny of the olive fruit. Taking these results into account, the expression levels of the olive *TDC* genes were studied in the same material to determine their possible involvement in the synthesis of the Ty and HTy derivatives. In general, it was observed (Figure 5) that the expression profiles of *OeTDC1* and *OeTDC2* during fruit ontogeny were very similar, with the average expression level of *OeTDC1* being consistently higher than that of *OeTDC2*. This difference in expression levels between the olive TDC genes averages around 8.5-fold but varies significantly between cultivars. This similarity in these profiles suggests similar regulation for both *TDC* genes. The expression profile closely mirrors the pattern observed for the content of the Ty and HTy derivatives [12], showing an intense expression of both genes at 3 WAF. However, in cultivars such as ‘Arbequina’ or ‘Menya’, the maximum expression level shifts slightly to 8 WAF. A different degree of decline in the expression levels of both genes was also observed during olive fruit ontogeny. Dividing this process into two halves of approximately 15 weeks each, the average expression level of *OeTDC1* drops about 130-fold in the second half of ontogeny compared to the first half. In contrast, *OeTDC2* decreases only about 15-fold in the second half.

Correlation analyses were performed between the content of the main groups of phenolic compounds in the fruits and the expression levels of the olive *TDC* genes, or their combined total (Table 2). As shown, Pearson correlation coefficients indicate moderate to high levels of correlation and strong significance (*p* < 0.001) across all comparisons between the content of Ty and HTy derivatives, or their combined total, and the expression levels of *OeTDC1*, *OeTDC2*, or their combined total. The computed Pearson coefficients for Ty derivatives are consistently slightly higher than those for HTy derivatives. The highest correlations were observed when considering the combined expression levels of both genes (*r* = 0.63 and 0.59 for Ty and HTy derivatives, respectively). Furthermore, the data from the ‘Dokkar’ cultivar were found to introduce a significant distortion in this correlation analysis, greatly affecting the results. When the analysis is performed with the exclusion of this cultivar, the Pearson coefficients rise significantly (*r* = 0.75 and 0.76 for the combined expression levels of both *TDC* genes and the content of Ty and HTy derivatives, respectively), while the remaining cultivars contribute similarly.

These results, together with the catalytic properties described above for the proteins encoded by the *OeTDC1* and *OeTDC2* genes, suggest that both genes are involved in the synthesis of Ty and HTy derivatives in the olive fruit and that they act jointly in a proportion according to their expression levels and the *k*_cat_ values of the proteins they encode.

## 3. Materials and Methods

### 3.1. Plant Material

Seven olive cultivars (*Olea europaea* L.), ‘Dokkar’, ‘Menya’, ‘Piñonera’, ‘Picual’, ‘Arbequina’, ‘Fishomi’, and ‘Abou kanani’, were studied, and they are characterized by a wide range of phenolic compound contents in both olive fruit and oil [12]. These olive cultivars were selected from a genetically diverse olive collection representative of the olive species (Core-36 olive core collection maintained at the World Olive Germplasm Bank, IFAPA Alameda del Obispo, Cordoba, Spain), based on their highly contrasting phenolic compound contents [5]. Two trees per cultivar were selected from the experimental orchards of the Instituto de la Grasa, grown in a 5 × 6 m spacing pattern, using drip irrigation and fertigation from flowering to full ripening. Fruit sampling was performed by hand from 3 to 20 weeks after flowering (WAF) during fruit development, with an additional sample taken at the ripening stage (R, turning stage, around 50% color, MI ~ 2.5). The mesocarp of the collected fruits (20–50 per sample) was frozen in liquid nitrogen and stored at −80 °C until RNA extraction.

### 3.2. In Silico Analysis of Putative Olive TDC Genes

Putative *TDC* genes involved in the biosynthesis of Ty and HTy phenolic derivatives in olive fruit were selected from those annotated as tyrosine and tyrosine/DOPA decarboxylase in a transcriptomic study involving olive cultivars with distinct phenolic contents (Table S1) [10]. These genes were annotated using Sma3s [34] and Blast2GO v5.2 [35] software against the olive genome database (OE6.OLIVEFAT, https://denovo.cnag.cat/olive_data, accessed on 19 December 2017).

Olive TDC sequences were aligned with known plant aromatic amino acid decarboxylases using the ClustalW algorithm, and the conserved domains were analyzed with the NCBI Conserved Domain Search (https://www.ncbi.nlm.nih.gov/cdd/, accessed on 22 March 2024) and the Pfam software (http://pfam.sanger.ac.uk/, accessed on 22 March 2024). A phylogenetic analysis of olive TDCs with plant AAADs (accession numbers in Table S2) was also conducted using the neighbor-joining method. In both cases, the results were visualized using the Geneious program (https://www.geneious.com, accessed on 27 October 2023).

The predicted isoelectric point (pI) and molecular mass of the studied proteins were calculated using ExPASy Compute pI/Mw (https://web.expasy.org/compute_pi/, accessed on 1 April 2024). The subcellular localizations of the proteins were predicted using the online DeepLoc-2.0 tool (http://www.cbs.dtu.dk/services/DeepLoc/, accessed on 4 June 2024) and TargetP2.0 (http://www.cbs.dtu.dk/services/TargetP/, accessed on 4 June 2024) was used to assess the presence of signal peptides at the N-terminus of the sequences.

### 3.3. cDNA Library Construction

cDNA libraries were prepared from the mesocarp of the olive fruit from the seven cultivars under study, sampled throughout different stages of development and ripening. The RNA was first extracted from three biological samples per cultivar/development stage using the Spectrum Plant Total RNA kit (STRN250, Sigma-Aldrich, St. Louis, MO, USA). The RNA concentration and integrity were determined using nanodrop photometric quantification (Thermo Scientific, Waltham, MA, USA). Finally, cDNA was synthesized from these RNA samples using the Ready-To-Go You-Prime First-Strand Beads kit (Cytiva, Little Chalfont, UK) and Oligo (dT) 18 Primer (Thermo Scientific, Waltham, MA, USA).

### 3.4. Gene Expression Analysis

Olive *TDC* gene expression analyses were performed in a CFX96 Touch System (Biorad, Hercules, CA, USA) by RT-qPCR using the prepared cDNA libraries, a pair of primers specific for each olive *TDC* (Table S3) and SYBR Green I (SsoAdvancedTM Universal SYBR^®^ Green Supermix, 1725271, Biorad, Hercules, CA, USA). The reaction mixture was first heated for 30 s at 95 °C. Next, it was subjected to 40 temperature/time cycles of 95 °C for 15 s; 15 s at 51 °C for *OeTDC1* and 54 °C for *OeTDC2*; and finally at 60 °C for 15 s. Expression levels were calculated using BioRad CFX Maestro 1.0 software (Biorad, Hercules, CA, USA) and the Pfaffl method [36]. The efficiencies for each primer pair were calculated using sequential dilutions of cDNA with the aid of GeNorm algorithm included in the BioRad CFX Maestro 1.0 software. Reference genes, including *Ser/Thr phosphatase 2A* (*OePP2A*), *glyceraldehyde-3-phosphate dehydrogenase* (*OeGAPDH*) and *elongation factor-1-alpha* (*OeEF1α*) (olive genome database annotation numbers OE6A097517, OE6A105640 and OE6A045598, https://denovo.cnag.cat/olive_data, accessed on 19 December 2017) were selected based on previous validation studies [37,38]. The specific primer pairs for these reference genes, as well as for *OeTDC1* and *OeTDC2* are described in Supplementary Material (Table S3). Two technical replicates were obtained from each sample, and statistical significance was determined using Tukey’s test at *p* < 0.05.

### 3.5. Heterologous Gene Expression and Purification of Recombinant Proteins

The coding sequences of the selected olive *OeTDC1* and *OeTDC2* genes (Olive Genome Database accession OE6A082511, GenBank PP534480, and OE6A073377, GenBank PP590793, respectively) were synthesized with *E. coli* codon optimization (GenScript) and cloned into a pET-28a(+)-TEV vector as NheI-SalI fragment. *E. coli* BL21(DE3) cells carrying plasmid pIZ227, which provide efficient repression of the synthesis of the T7 RNA polymerase from the lacUV5 promoter [39] and containing the *OeTDC1* and *OeTDC2* constructs were grown at 37 °C to an OD_600_ of 0.6 in Luria–Bertani (LB) medium supplemented with 0.5 M NaCl. Expression of these genes was then induced by adding 0.4 mM isopropyl-β-D-thiogalactoside (IPTG) to the culture medium. After 18 h at 18 °C, cells were harvested by centrifugation. Cells were then washed with PBS buffer containing 137 mM NaCl, 2.7 mM KCl, 10 mM Na_2_HPO_4_, and 1.8 mM KH_2_PO_4_, resuspended in lysis buffer consisting of 50 mM Tris-HCl, pH 8.0, 0.5 M NaCl, 0.2 mM PLP, 0.5 mM DTT, 40 mM imidazole, 1 mM PMSF, 1% Protease Inhibitor Cocktail (Sigma-Aldrich, St. Louis, MO, USA), and 10% glycerol.

To obtain the recombinant proteins, cells were lysed by sonication, clarified by centrifugation, and purified using nickel-Sepharose affinity chromatography with His GraviTrap columns (GE Healthcare, Chicago, IL, USA). The recombinant protein bound to nickel-Sepharose was washed with six column volumes of buffer containing 50 mM Tris-HCl, pH 7.4, 0.5 M NaCl, 0.2 mM PLP, 0.5 mM DTT and 40 mM imidazole (washing buffer). It was then eluted with two column volumes of elution buffer, which had the same composition as washing buffer but with 500 mM imidazole. Subsequently, imidazole was removed from the recombinant protein solution using a PD-10 column (Sephadex G-25, GE Healthcare, Chicago, IL, USA), concentrated by means of a Vivaspin centrifugal concentrator (MWCO 30 kDa, Merck, Darmstadt, Germany) and stored at 4 °C in storage buffer (20 mM Tri-HCl, pH 8.0, 25 mM NaCl, 0.2 mM PLP, 0.5 mM DTT and 10% glycerol). Finally, the degree of purity of the isolated recombinant protein was measured by SDS-PAGE and densitometry analysis (ChemiDoc Imaging System, Biorad, Hercules, CA, USA). The concentration of proteins was determined using the Bio-Rad protein assay kit with bovine serum albumin as a standard.

In the experiments used to determine the necessity of PLP for the active form of the proteins, the composition of the lysis buffer was the same but without PLP. Similarly, the buffers used later in the purification of the recombinant enzymes were the same as those described but without PLP.

### 3.6. Tyrosine Decarboxylase Activity Assay

The enzymatic activity assays were conducted by incubating the recombinant olive TDC proteins (92 and 205 ng of recombinant *Oe*TDC1 and *Oe*TDC2, respectively) for 15 min at 40 °C with different amino acids (DOPA, tyrosine, tryptophan, phenylalanine) in a wide concentration range (0.05–5 mM). The reaction medium consisted of 200 µL of reaction buffer (50 mM Tris-HCl, pH 8, 200 µM PLP). Reactions were stopped by adding 200 µL of 0.8 M formic acid to the reaction medium. The mixture was then centrifuged, and the supernatant was analyzed by high-performance liquid chromatography (HPLC).

Analysis of substrates and reaction products was performed on a Beckman Coulter HPLC system equipped with a DAD System Gold 168 detector and a SunShell C18 column (4.6 × 250 mm, particle size 3.5 μm. ChromaNik Technologies Inc., Osaka, Japan). The mobile phase was a ternary mixture of 0.5% phosphoric acid (mobile phase A) and methanol–acetonitrile (1:1) (mobile phase B) that allowed separation of substrates and reaction products. When using tyrosine and DOPA as substrates, the analysis started at 0% B and increased to 10.5% in 3 min, and it became isocratic at 10.5% B. The detection of the respective reaction products, tyramine (R_t_ = 6.3 min) and dopamine (R_t_ = 5.2 min), was performed at 280 nm. When tryptophan was used as a substrate, the analysis started at 0% B and increased to 30% in 15 min and the detection of tryptamine was performed at 276 nm (R_t_ = 13.1 min). When phenylalanine was used as a substrate, the analysis started at 20% and increased to 37.5% in 5 min, then isocratic at 37.5% B. Phenylethylamine was detected at 258 nm (R_t_ = 3.5 min). The amount of reaction product generated in the reactions was measured from previously generated curves with commercial standards corresponding to each of the possible reaction products (tyramine, dopamine, 2-phenylethylamine, tryptamine) as shown in Figure S4. The identity of the reaction products was determined by comparing the retention time with that of the commercial standards, UV spectrometry, and mass spectrometry using a Dionex Ultimate 3000 RS U-HPLC/ESI-qTOF-HRMS liquid chromatograph (Thermo Fisher Scientific, Waltham, MA, USA) equipped with a similar column and elution program, except that the mobile phase used 0.1% formic acid instead of 0.5% phosphoric acid.

### 3.7. Statistical Analysis

Statistical analysis was performed using Excel 2016 and STATISTICA 8.0 (Statsoft Inc., Tulsa, OK, USA). Pearson’s correlation coefficients were calculated to assess the relationship between the gene expression levels of olive *TDC* genes and the content of tyrosol and hydroxytyrosol derivatives in olive fruit [12]. A *p*-value of <0.001 was considered to be statistically significant.

## 4. Conclusions

The data obtained on the molecular characteristics of the olive *TDC* genes are consistent with their participation in the biosynthetic process that leads to the production of Ty and HTy, as well as their secoiridoid derivatives, which are present in olive fruit and VOO. The catalytic properties of the recombinant proteins produced corroborate these molecular characteristics, demonstrating that both olive TDCs accept exclusively aromatic amino acids with phenolic side chains—tyrosine and DOPA—as substrates to produce tyramine and dopamine, respectively. These were the exclusive reaction products, as there was no evidence of AAS-type activity. Both proteins exhibited higher affinity for DOPA than for tyrosine and the catalytic efficiency of both proteins was higher with DOPA as the substrate. These data align with the greater presence of HTy derivatives compared to Ty derivatives in the fruits and oils, a common characteristic across the entire olive species. Although correlation studies show quite similar values between the expression levels of both olive *TDC* genes and the content of Ty and HTy derivatives, the higher expression levels observed for the *OeTDC1* gene in the studied cultivars suggest a greater role for this gene in the synthesis of Ty and HTy derivatives in olive fruit and, consequently, in virgin olive oil. These results could be valuable for identifying molecular markers in marker-assisted olive breeding programs aimed at developing new cultivars with enhanced phenolic profiles and improved functional quality.

## Figures and Tables

**Table 1:** Kinetic parameters for olive TDCs towards tyrosine and DOPA.

	Substrate	Optimum pH	Optimum Temp. (°C)	*K*m (mM)	*V*_max_ (nkat mg^−1^)	*k*_cat_ (s^−1^)	*k*_cat_/*K*m (s^−1^ M^−1^)
*Oe*TDC1	Tyrosine	8.0	40	1.90	206	12.79	6717
*Oe*TDC1	DOPA			0.34	68	3.82	11,335
*Oe*TDC2	Tyrosine	8.0	40	1.29	425	23.98	18,589
*Oe*TDC2	DOPA			0.46	257	14.50	31,522

**Table 2:** Pearson correlation coefficients (*r*) between the expression levels of olive *TDC* genes and the content of the main groups of phenolics in the fruit of seven olive cultivars with contrasting phenolics contents [12].

	*OeTDC2*	*OeTDC1*	*OeTDC1* + *OeTDC2*
HTy-Derivatives	0.49	0.51	0.59
Ty-Derivatives	0.51	0.56	0.63
Ty + HTy-Derivatives	0.50	0.53	0.61

## Data Availability

Data are contained within the article.

## Supplementary Materials

The following supporting information can be downloaded at https://www.mdpi.com/article/10.3390/ijms252010892/s1.

## References

[1] Estruch R., Ros E., Salas-Salvadó J., Covas M.I., Corella D., Arós F., Gómez-Gracia E., Ruiz-Gutiérrez V., Fiol M., Lapetra J. (2018). Primary prevention of cardiovascular disease with a Mediterranean diet supplemented with extra-virgin olive oil or nuts. N. Engl. J. Med..

[2] Infante R., Infante M., Pastore D., Pacifici F., Chiereghin F., Malatesta G., Donadel G., Tesauro M., Della-Morte D. (2023). An Appraisal of the oleocanthal-rich extra virgin olive oil (EVOO) and its potential anticancer and neuroprotective properties. Int. J. Mol. Sci..

[3] Wang R., Ganbold M., Ferdousi F., Tominaga K., Isoda H.A. (2023). Rare olive compound oleacein improves lipid and glucose metabolism, and inflammatory functions: A comprehensive whole-genome transcriptomics analysis in adipocytes differentiated from healthy and diabetic adipose stem cells. Int. J. Mol. Sci..

[4] Konstantinidou V., Covas M.I., Muñoz-Aguayo D., Khymenets O., de La Torre R., Saez G., del Carmen Tormos M., Toledo E., Marti A., Ruiz-Gutiérrez V. (2010). In vivo nutrigenomic effects of VOO polyphenols within the frame of the Mediterranean diet: A randomized trial. FASEB J..

[5] García-Rodríguez R., Belaj A., Romero-Segura C., Sanz C., Pérez A.G. (2017). Exploration of genetic resources to improve the functional quality of virgin olive oil. J. Funct. Foods.

[6] Pérez A.G., León L., Sanz C., de la Rosa R. (2018). Fruit phenolic profiling: A new selection criterion in olive breeding programs. Front. Plant Sci..

[7] Pérez A.G., León L., Pascual M., de la Rosa R., Belaj A., Sanz C. (2019). Analysis of olive (*Olea europaea* L.) genetic resources in relation to the content of vitamin E in virgin olive oil. Antioxidants.

[8] Savarese M., de Marco E., Sacchi R. (2007). Characterization of phenolic extracts from olives (*Olea europaea* cv. Pisciottana) by electrospray ionization mass spectrometry. Food Chem..

[9] Romero-Segura C., García-Rodríguez R., Sánchez-Ortiz A., Sanz C., Pérez A.G. (2012). The role of olive beta-glucosidase in shaping the phenolic profile of virgin olive oil. Food Res. Int..

[10] Sánchez R., García-Vico L., Sanz C., Pérez A. (2019). An aromatic aldehyde synthase controls the synthesis of hydroxytyrosol derivatives present in virgin olive oil. Antioxidants.

[11] Lan X., Chang K., Zeng L., Liu X., Qiu F., Zheng W., Quan H., Liao Z., Chen M., Huang W. (2013). Engineering salidroside biosynthetic pathway in hairy root cultures of *Rhodiola crenulata* based on metabolic characterization of tyrosine decarboxylase. PLoS ONE.

[12] Luaces P., Expósito J., Benabal P., Pascual M., Sanz C., Pérez A.G. (2024). Accumulation patterns of metabolites responsible for the functional quality of virgin olive oil during olive fruit ontogeny. Antioxidants.

[13] Facchini P.J., Huber-Allanach K.L., Tari L.W. (2000). Plant aromatic L-amino acid decarboxylases: Evolution, biochemistry, regulation, and metabolic engineering applications. Phytochemistry.

[14] György Z., Jaakola L., Neubauer P., Hohtola A. (2009). Isolation and genotype-dependent, organ-specific expression analysis of a *Rhodiola rosea* cDNA encoding tyrosine decarboxylase. J. Plant Physiol..

[15] Gou Y., Li T., Wang Y. (2024). Active-site oxygen accessibility and catalytic loop dynamics of plant aromatic amino acid decarboxylases from molecular simulations. Biochemistry.

[16] Wang H., Yu J., Satoh Y., Nakagawa Y., Tanaka R., Kato K., Yao M. (2020). Crystal structures clarify cofactor binding of plant tyrosine decarboxylase. Biochem. Biophys. Res. Commun..

[17] Park S.U., Johnson A.G., Penzes-Yost C., Facchini P.J. (1999). Analysis of promoters from tyrosine/dihydroxyphenylalanine decarboxylase and berberine bridge enzyme genes involved in benzylisoquinoline alkaloid biosynthesis in opium poppy. Plant Mol. Biol..

[18] Kawalleck P., Keller H., Hahlbrock K., Scheel D., Somssich I.E. (1993). A pathogen-responsive gene of parsley encodes tyrosine decarboxylase. J. Biol. Chem..

[19] Saimaru H., Orihara Y. (2010). Biosynthesis of acteoside in cultured cells of *Olea europaea*. J. Nat. Med..

[20] Yang Y.H., Yang M.R., Zhu J.Y., Dong K.W., Yi Y.J., Li R.F., Zeng L., Zhang C.F. (2022). Functional characterization of tyrosine decarboxylase genes that contribute to acteoside biosynthesis in *Rehmannia glutinosa*. Planta.

[21] Almagro-Armenteros J., Sønderby C., Sønderby S., Nielsen H., Winther O. (2017). DeepLoc: Prediction of protein subcellular localization using deep learning. Bioinformatics.

[22] Almagro-Armenteros J., Salvatore M., Emanuelsson O., Winther O., von Heijne G., Elofsson A., Nielsen H. (2019). Detecting sequence signals in targeting peptides using deep learning. Life Sci. Alliance.

[23] Kaminaga Y., Schnepp J., Peel G., Kish C.M., Ben-Nissan G., Weiss D., Orlova I., Lavie O., Rhodes D., Wood K. (2006). Plant phenylacetaldehyde synthase is a bifunctional homotetrameric enzyme that catalyzes phenylalanine decarboxylation and oxidation. J. Biol. Chem..

[24] Torrens-Spence M.P., Liu P., Ding H., Harich K., Gillaspy G., Li J. (2013). Biochemical evaluation of the decarboxylation and decarboxylation-deamination activities of plant aromatic amino acid decarboxylases. J. Biol. Chem..

[25] Torrens-Spence M.P., Lazear M., von Guggenberg R., Ding H., Li J. (2014). Investigation of a substrate-specifying residue within *Papaver somniferum* and *Catharanthus roseus* aromatic amino acid decarboxylases. Phytochemistry.

[26] Facchini P.J., De Luca V. (1995). Expression in *Escherichia coli* and partial characterization of two tyrosine/dopa decarboxylases from opium poppy. Phytochemistry.

[27] Zhu H., Xu G., Zhang K., Kong X., Han R., Zhou J., Ni Y. (2016). Crystal structure of tyrosine decarboxylase and identification of key residues involved in conformational swing and substrate binding. Sci. Rep..

[28] Torrens-Spence M.P., Chiang Y.-C., Smith T., Vicent M.A., Wang Y., Weng J.-K. (2020). Structural basis for divergent and convergent evolution of catalytic machineries in plant aromatic amino acid decarboxylase proteins. Proc. Natl. Acad. Sci. USA.

[29] Ishii S., Mizuguchi H., Nishino J., Hayashi H., Kagamiyama H. (1996). Functionally important residues of aromatic l-amino acid decarboxylase probed by sequence alignment and site-directed mutagenesis. J. Biochem..

[30] Marques I.A., Brodelius P.E. (1988). Elicitor-induced l-tyrosine decarboxylase from plant cell suspension cultures 1: I. Induction and purification. Plant Physiol..

[31] Torrens-Spence M.P., Pluskal T., Li F.-S., Carballo V., Weng J.-K. (2018). Complete pathway elucidation and heterologous reconstitution of rhodiola salidroside biosynthesis. Mol. Plant.

[32] Lehmann T., Pollmann T. (2009). Gene expression and characterization of a stress induced tyrosine decarboxylase from *Arabidopsis thaliana*. FEBS Lett..

[33] Torrens-Spence M.P., Gillaspy G., Zhao B., Harich K., White R.H., Li J. (2012). Biochemical evaluation of a parsley tyrosine decarboxylase results in a novel 4-hydroxyphenylacetaldehyde synthase enzyme. Biochem. Biophys. Res. Commun..

[34] Muñoz-Merida A., Viguera E., Claros M.G., Trelles O., Perez-Pulido A.J. (2014). Sma3s: A three-step modular annotator for large sequence datasets. DNA Res..

[35] Conesa A., Götz S., García-Gómez J.M., Terol J., Talón M., Robles M. (2005). Blast2GO: A universal tool for annotation, visualization and analysis in functional genomics research. Bioinformatics.

[36] Pfaffl M.W. (2001). A new mathematical model for relative quantification in real-time RT-PCR. Nucleic Acids Res..

[37] Nonis A., Vezzaro A., Ruperti B. (2012). Evaluation of RNA extraction methods and identification of putative reference genes for real-time quantitative polymerase chain reaction expression studies on olive (*Olea europaea* L.) fruits. J. Agric. Food Chem..

[38] Ben ali S., Guasmi F., Mohamed M.B., Benhaj K., Boussora F., Triki T., Kammoun N.G. (2018). Identification of internal control genes for gene expression studies in olive mesocarp tissue during fruit ripening. S. Afr. J. Bot..

[39] Govantes F., Molina-López J.A., Santero E. (1996). Mechanism of coordinated synthesis of the antagonistic regulatory proteins NifL and NifA of *Klebsiella pneumoniae*. J. Bacteriol..

