# Supplementary material for: Functional and Physiological Characterization of Tyrosine Decarboxylases from Olea europaea L. Involved in the Synthesis of the Main Phenolics in Olive Fruit and Virgin Olive Oil
Source: Int J Mol Sci. 2024 Oct 10;25(20):10892. doi: 10.3390/ijms252010892 (PMC11507401; doi:10.3390/ijms252010892)
Supplement: Supplementary file 1 [file ijms-25-10892-s001.zip › ijms-3144519-supplementary.pdf]

Table S1. Annotation (Sma3s and Blast2GOPro softwares) and mean expression levels (FPKM) of putative tyrosine decarboxylase transcripts (CNAG olive genome database, OE6.OLIVEFAT) in ripening fruits (22-25 weeks after flowering) of seven olive cultivars (Dokkar, Menya, Piñonera, Picual, Klon, Fishomi and Abou kanani).

**Transcript**	***OE6A000744***	***OE6A082511***	***OE6A073377***	***OE6A011752***	***OE6A094428***
**Gene name**	*OeAAS*	*OeTDC1*	*OeTDC2*	-	-
**Sma3s annotation**	Pyridoxal phosphate-dependent decarboxylase	Putative tyrosine decarboxylase	Putative tyrosine decarboxylase	Pyridoxal phosphate-dependent decarboxylase	Tryptophan decarboxylase
**Blast2GOPro annotation**	Tyrosine decarboxylase 1	Tyrosine DOPA decarboxylase 1-like	Tyrosine DOPA decarboxylase 1-like	Tyrosine DOPA decarboxylase 1-like	Tyrosine DOPA decarboxylase 1-like
**Mean expression**	592	1011	253	0	4

Table S2. Protein sequences used for the phylogenetic analysis shown in Figure 3.

Protein	Plant species	GenBank Accession
*Oe*TyrDC1	*Olea europaea*	PP534480
*Oe*TyrDC2	*Olea europaea*	PP590793
*Ac*TyrDC	*Ananas comosus*	OAY76026
*Ac*TyrDC	*Aristplochia contorta*	ABJ16446
*Am*TyrDC	*Argemone mexicana*	ACJ76782
*At*PAAS	*Arabidopsis thaliana*	Q8RY79
*At*TyDC2	*Arabidopsis thaliana*	NP_001078461
*Bd*TyDC	*Brachypodium distachyon*	XP_3569907.1
*Ca*TrpDC1	*Camptotheca acuminata*	AAB39708
*Ca*TrpDC1	*Capsicum annuum*	ACN62127
*Ca*TrpDC2	*Camptotheca acuminata*	AAB39709
*Ca*TyrDC2	*Capsicum annuum*	XP_016541857.1
*Cr*TrpDC	*Catharanthus roseus*	P17770
*Cr*TyrDC1	*Cistrus reshni*	ACX29990
*Cs*TyrDC	*Citrus sinensis*	XP_024953754.2
*Gm*TyrDC2	*Glycine max*	XP_006576967.1
*Kn*AAS	*Klebsormidium nitens*	GAQ86385.1
*La*TyrDC1	*Lycoris aurea*	AYH64864
*Lr*TyrDC2	*Lycoris radiata*	QXF78538.1
*Md*TyrDC	*Malus domestica*	XP_008358473
*Nn*TyrDC	*Nelumbo nucifera*	XP_010245170
*Nt*TyrDC	*Nicotiana tabacum*	XP_016449301.1
*Oe*AAS	*Olea europaea*	QJA07379.1
*Op*TrpDC	*Ophiorrhiza pumila*	BAC41515.1
*Os*TrpDC	*Oryza sativa*	XP_015648701.1
*Os*TyrDC	*Oryza sativa*	015633932.1
*Pb*TyrDC	*Pyrus bretschneideri*	XP_009364243
*Pc*AAS	*Petrosilum crispum*	Q06086.1
*Pc*TyrDC4	*Petroselinum crispum*	AAA33863
*Ph*AAS	*Petunia hibrida*	ABB72475.1
*Pp*TyrDC	*Prunus persica*	XP_007213045.2
*Ps*TyrDC2	*Papaver somniferum*	P54769
*Ps*TyrDC	*Papaver somniferum*	AAC61842.1 1
*Pt*TyrDC	*Populus trichocarpa*	QBL52493.1
*Rc*TyrDC	*Rhodiola crenulata*	AFN89854.1
*Rg*TyDC1	*Rehmannia glutinosa*	ULR57004
*Rg*TyDC2	*Rehmannia glutinosa*	UKS50408
*Rg*TyDC3	*Rehmannia glutinosa*	ULR57005
*Rg*TyDC4	*Rehmannia glutinosa*	ULR57006
*Rh*AAS	*Rosa hybrid*	ABB04522.1
*Rr*AAS	*Rhodiola rosea*	AUI41112.1
*Rs*TyrDC	*Rhodiola sachalinensis*	ABF06560
*Si*TyrDC	*Sesamun indicum*	XP_011096642.1
*St*TyrDC	*Solanum tuberosa*	AHI16968.1
*Ta*TDC	*Triticum aestivum*	CDM81842
*Tc*TyrDC	*Theobroma cacao*	EOX96928
*Tf*TyrDC	*Thalictrum flavum*	AAG60665
*Tt*Tyr-DOPADC	*Thralictrum thalictroides*	KAAF5198316
*Zm*TyrDC	*Zea mays*	ACG46884.1
*Zm*TyrDC AAS	*Zostera marina*	KMZ74011.1

Table S3. Oligonucleotides used for RT-qPCR expression analysis of *OeTDC1* (GenBank PP534480) and *OeTDC2* (GenBank PP590793).

Name	Sequence (5´-3´)
q*Oe*TDC1-F	ACCCTTACAGAAAACAGGCA
q*Oe*TDC1-R	TTTTTGTCCATCTTCATCTTC
q*Oe*TDC2-F	ACCCTTACAGAAAACAGGCA
q*Oe*TDC2-R	CGCAAACTAAAATGGACAAAAGCA
q*Oe*EF1α-F	TGCTCTATCTGGATTGCCATT
q*Oe*EF1α-R	TCAAATGCCACCATGACTTC
q*Oe*GAPDH-F	TGAGATGCTGCACAATGGTT
q*Oe*GAPDH-R	CACGATAGGCTTACGCAACA
q*Oe*PP2A-F	CTCGCCTGAAAACGAAAGAC
q*Oe*PP2A-R	CACAAAGCAGACCAAAACCA

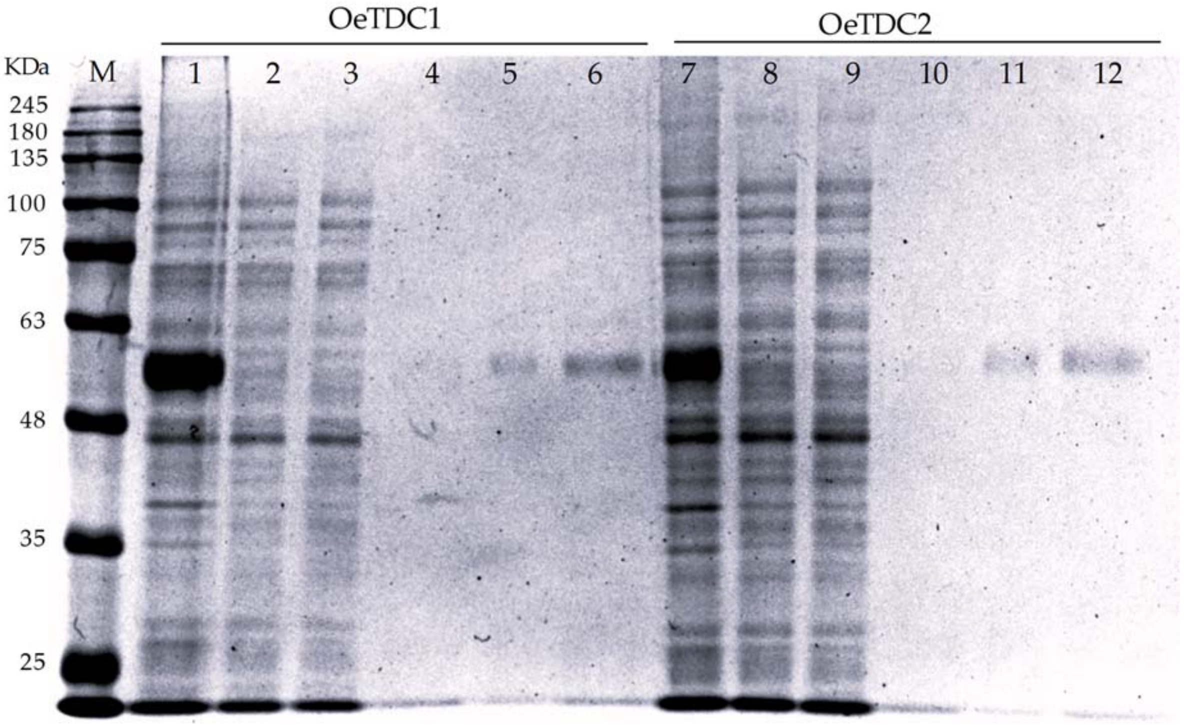

Figure S1. SDS-PAGE followed by a Coomassie blue staining of representative purifications of olive TDC recombinant proteins. M: molecular weight protein ladder; 1, *Oe*TDC1 induced sample; 2, BL21(DE3) pIZ227 *Oe*TDC1 control sample; 3, total *Oe*TDC1 induced sample eluted from Ni-Sepharose column; 4, Ni-Sepharose *Oe*TDC1 elution, fraction 1 (0.5 mL); 5, Ni-Sepharose *Oe*TDC1 elution, fraction 2 (2.5 mL); 6, Ni-Sepharose *Oe*TDC1 elution, re-eluted fraction 2; 7, *Oe*TDC2 induced sample; 8, BL21(DE3) pIZ227 *Oe*TDC2 control sample; 9, total *Oe*TDC2 induced sample eluted from Ni-Sepharose column; 10, Ni-Sepharose *Oe*TDC2 elution, fraction 1 (0.5 mL); 11, Ni-Sepharose *Oe*TDC2 elution, fraction 2 (2.5 mL); 12, Ni-Sepharose *Oe*TDC2 elution, re-eluted fraction 2.

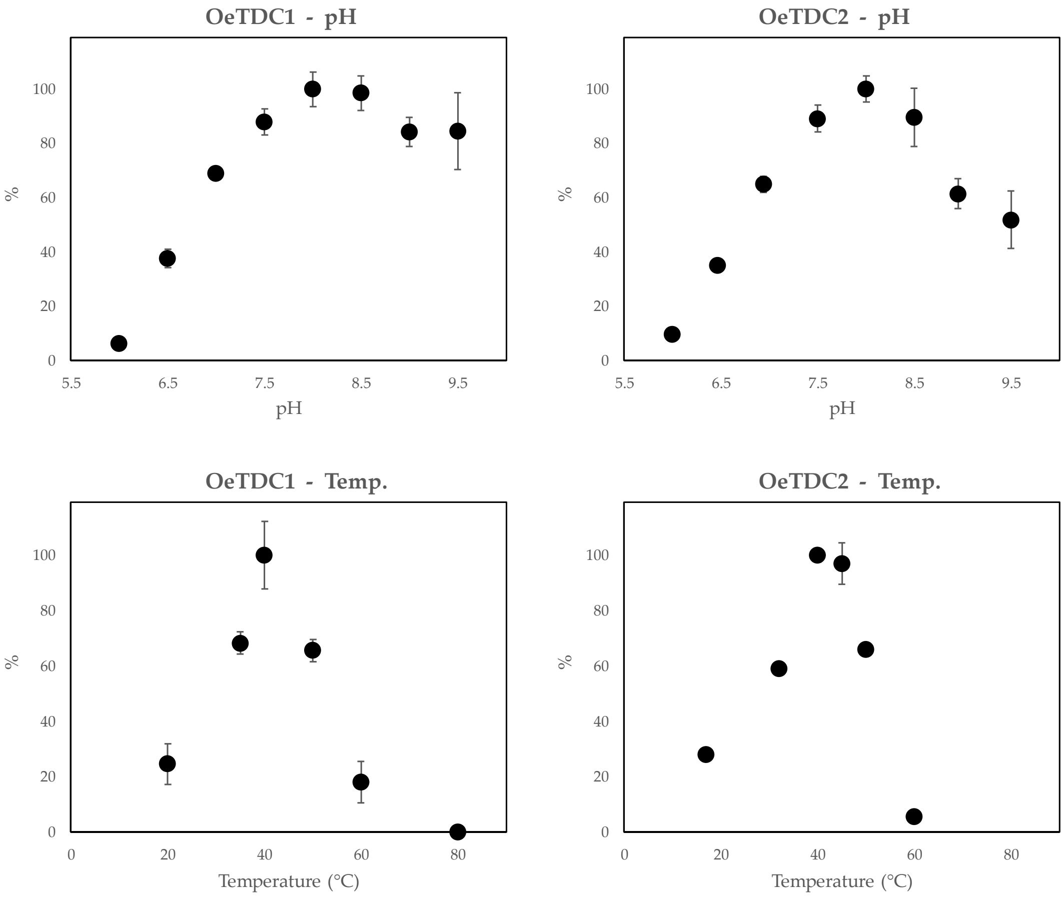

Figure S2. Effect of pH and temperature in the reaction medium for *Oe*TDC1 and *Oe*TDC2 using tyrosine as substrate.

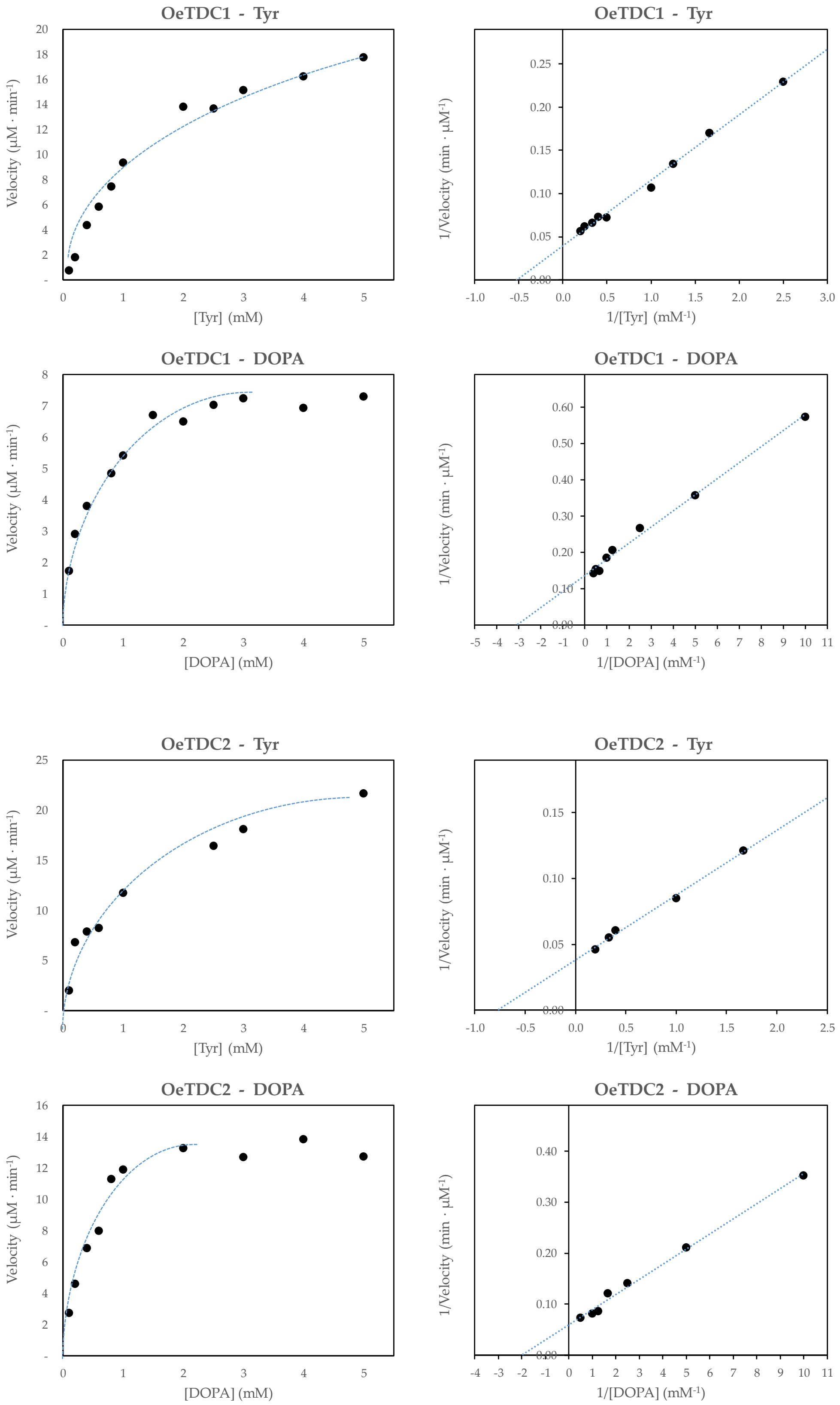

Figure S3. Determination of catalytic properties of *Oe*TDC1 and *Oe*TDC2 for tyrosine and DOPA. Olive TDCs were incubated at different concentrations of tyrosine or DOPA for 15 min at 40 °C. Activity was determined by analyzing the amount of reaction product by HPLC.

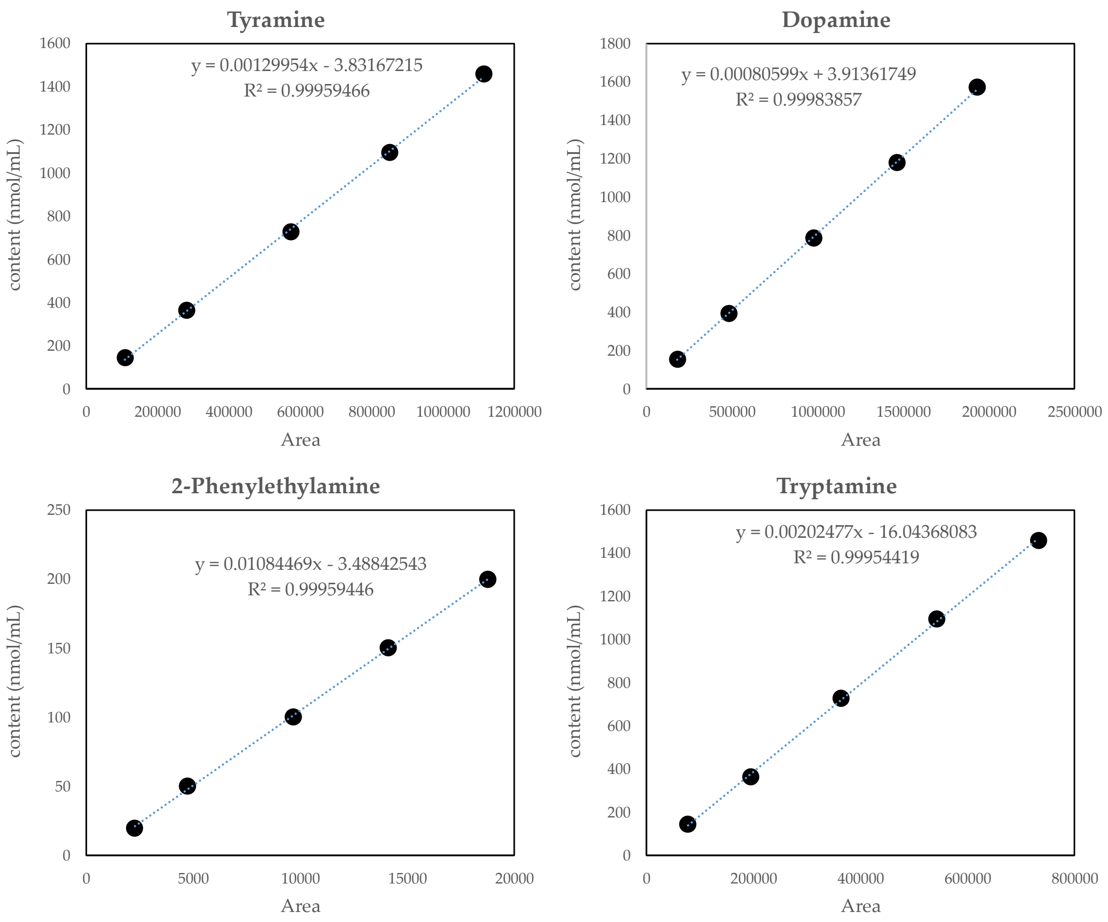

Figure S4. Calibration curves for the possible reaction products of *Oe*TDC1 and *Oe*TDC2. Tyramine and dopamine were monitored at 280 nm, 2-phynylethylamine at 258 nm, and tryptamine at 276 nm.
